# Metabolic engineering for the production of shikimic acid in an evolved *Escherichia coli *strain lacking the phosphoenolpyruvate: carbohydrate phosphotransferase system

**DOI:** 10.1186/1475-2859-9-21

**Published:** 2010-04-12

**Authors:** Adelfo Escalante, Rocío Calderón, Araceli Valdivia, Ramón de Anda, Georgina Hernández, Octavio T Ramírez, Guillermo Gosset, Francisco Bolívar

**Affiliations:** 1 Departamento de Ingeniería Celular y Biocatálisis, Instituto de Biotecnología, Universidad Nacional Autónoma de México (UNAM). Av. Universidad 2001, Col. Chamilpa, Cuernavaca, Morelos, 62210, México; 2 Departamento de Medicina Molecular y Bioprocesos, Instituto de Biotecnología, Universidad Nacional Autónoma de México (UNAM). Av. Universidad 2001, Col. Chamilpa, Cuernavaca, Morelos, 62210, México

## Abstract

**Background:**

Shikimic acid (SA) is utilized in the synthesis of oseltamivir-phosphate, an anti-influenza drug. In this work, metabolic engineering approaches were employed to produce SA in *Escherichia coli *strains derived from an evolved strain (PB12) lacking the phosphoenolpyruvate:carbohydrate phosphotransferase system (PTS^-^) but with capacity to grow on glucose. Derivatives of PB12 strain were constructed to determine the effects of inactivating *aroK*, *aroL*, *pykF or pykA *and the expression of plasmid-coded genes *aroG*^fbr^, *tktA, aroB *and *aroE*, on SA synthesis.

**Results:**

Batch cultures were performed to evaluate the effects of genetic modifications on growth, glucose consumption, and aromatic intermediate production. All derivatives showed a two-phase growth behavior with initial high specific growth rate (*μ*) and specific glucose consumption rate (*qs*), but low level production of aromatic intermediates. During the second growth phase the *μ *decreased, whereas aromatic intermediate production reached its maximum. The double *aroK*^- ^*aroL*^- ^mutant expressing plasmid-coded genes (strain PB12.SA22) accumulated SA up to 7 g/L with a yield of SA on glucose of 0.29 mol/mol and a total aromatic compound yield (TACY) of 0.38 mol/mol. Single inactivation of *pykF or pykA *was performed in PB12.SA22 strain. Inactivation of *pykF *caused a decrease in *μ*, *qs*, SA production, and yield; whereas TACY increased by 33% (0.5 mol/mol).

**Conclusions:**

The effect of increased availability of carbon metabolites, their channeling into the synthesis of aromatic intermediates, and disruption of the SA pathway on SA production was studied. Inactivation of both *aroK *and *aroL*, and transformation with plasmid-coded genes resulted in the accumulation of SA up to 7 g/L with a yield on glucose of 0.29 mol/mol PB12.SA22, which represents the highest reported yield. The *pykF *and *pykA *genes were inactivated in strain PB12.SA22 to increase the production of aromatic compounds in the PTS^- ^background. Results indicate differential roles of Pyk isoenzymes on growth and aromatic compound production. This study demonstrated for the first time the simultaneous inactivation of PTS and *pykF *as part of a strategy to improve SA production and its aromatic precursors in *E. coli*, with a resulting high yield of aromatic compounds on glucose of 0.5 mol/mol.

## Background

**Figure 1:**
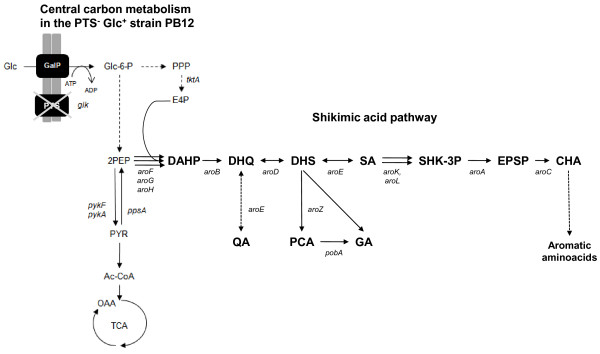
**Central carbon metabolism and shikimic acid pathways in *E. coli *PB12 strain lacking the PTS**. Glucose transport and phosphorylation are performed by GalP and Glk, respectively [27]. Abbreviations: Glc, glucose; GalP, galactose permease; Glc-6-P, glucose-6-P; Glk, glucokinase; PEP, phosphoenol pyruvate; PYR, pyruvate; Ac-CoA, acetyl coenzyme-A; TCA, tricarboxylic acid cycle; OAA, oxaloacetate; PPP, pentose phosphate pathway; E4P, erythrose-4-P; DAHP, 3-deoxy-D-*arabino*heptulosonate-7-P; DHQ, 3-dehydroquinic acid; DHS, 3-dehydroshikimic acid; SA, shikimic acid; S3P, shikimate-3-P; EPSP, 5-enolpyruvylshikimate-3-phosphate; CHA, chorismic acid; QA, quinic acid; PCA, protocatehuic acid; GA, gallic acid. Genes and coded enzymes: *tktA*, transketolase I;*pykF*, pyruvate kinase I;*pykA*, pyruvate kinase II;*ppsA*, phosphoenolpyruvate synthase; *aroF, aroG, aroH*, DAHP synthase isoenzymes F, G and H, respectively; *aroB*, DHQ synthase; *aroD*, DHQ dehydratase; *aroE*, shikimate dehydrogenase; *aroK*, shikimate kinase I; *aroL*, shikimate kinase II; *aroA*, EPSP synthase; *aroC*, chorismate synthase; *aroZ*, dehydroshikimate dehydratase; *pobA*, *p-*hydroxy-benzoate hydroxylase [32]. Continuous arrows represent unique reactions catalyzed by one or more enzymes; dotted lines or arrows represent two or more enzymatic reactions or incomplete characterized reactions.

The shikimic acid (SA) pathway is the common route leading to the biosynthesis of aromatic compounds in bacteria and in several eukaryotic organisms such as ascomycetes fungi, apicomplexans, and plants [1,2]. In *Escherichia coli*, the first step in this pathway is the condensation of the central carbon metabolism (CCM) intermediates phosphoenol pyruvate (PEP) and erythrose 4-phosphate (E4P) into 3-deoxy-D-*arabino*heptulosonate 7-phosphate (DAHP) by the DAHP synthase (DAHPS) isoenzymes AroF, AroG, and AroH, coded respectively by the *aroF, aroG *and *aroH *genes (Figure 1).

DAHP is converted to 3-dehydroquinate (DHQ) by dehydroquinate synthase, coded by *aroB*. DHQ dehydratase, coded by *aroD*, catalyzes the transformation of DHQ into 3-dehydroshikimic acid (DHS). This compound is reduced to SA by the shikimate dehydrogenase, coded by *aroE*. In turn, SA is transformed to shikimate-3-P (SHK-3P) by the shikimate kinase isoenzymes I and II, coded by the *aroK *and *aroL *genes, respectively; SHK-3P is then transformed to chorismic acid (CHA) (Figure 1).

SA is used as the precursor for the synthesis of a large number of chemicals [3-5] and nowadays has gained importance as the starting compound for the chemical synthesis of the neuraminidase inhibitor oseltamivir phosphate ((3R,4R,5S)-4-acetylamino-5-amino-3 (1-ethylpropoxy)-1-cyclohexene-1-carboxylic acid, ethyl ester phosphate (1:1)) known as Tamiflu^® ^and produced by Roche Pharmaceuticals. This compound is currently employed as an antiviral drug for the treatment of both common seasonal influenza A and B virus infections [6,7] and for the treatment of both the avian virus type H5N1 and A/H1N1 influenza infections. The latter has been considered a new pandemic [8,9]. It has been estimated that in the case of a global pandemic of influenza, the present capacity of Tamiflu^® ^production could be insufficient to protect large populations, particularly in developing countries [7,8]. Thus, alternative biotechnological strategies with engineered strains to produce SA have gained relevance.

Several metabolic engineering approaches have been developed to obtain SA from *E. coli *by biotechnological processes as an alternative to its limited and costly extraction procedures from plants such as *Illicium anisatum *or *I. verum *[3,5,9-11]. Previously developed approaches involve *E. coli *derivatives with several genetic modifications in the CCM and SA pathways. CCM modifications comprise inactivation of the PTS operon (*ptsHIcrr*), expression of non-PTS glucose transporters like glucose facilitators and transformation with plasmids carrying the *tktA *and *ppsA *genes, coding for transketolase I and PEP synthase, respectively, to increase the availability of intermediates E4P and PEP, respectively [3,4,12-18]. The main modifications in the SA pathway include the partial or total blockage of the SA flux into CHA. This has been achieved by decreasing or completely eliminating the synthesis of SHK-3P -by inactivating *aroK *and a*roL *genes- with the subsequent SA accumulation (Figure 1). These modifications are commonly complemented with the transformation of plasmid-coded feedback resistant (fbr) AroF or AroG proteins (AroF^fbr ^and AroG^fbr^, respectively), required to avoid possible feedback inhibition in the first step of the aromatic pathway catalyzed by DAHPS isoenzymes. The rate-limiting enzyme DHQ synthase, and shikimate dehydrogenase, which is feedback inhibited by SA [3,15], catalyze two reactions that can be improved with the goal of increasing the synthesis of SA. It has been proposed that high extracellular SA concentration drives the transport of this compound into the cells by the SA transporter ShiA (*shiA*). Higher intracellular SA accumulation reverts the reaction catalyzed by *aroE *to synthesize DHS, resulting in "hydroaromatic equilibration" and by-productby formation, such as quinic (QA) and gallic acids (GA) (Figure 1). Inactivation of the ShiA transporter has been used as a strategy to reduce the intracellular accumulation of DHS, QA, and GA [3,4,15,19,20]. Engineered *E. coli *strains with several of the genetic modifications described above have been successfully applied to produce 71 g/L of SA with a yield of 0.27 mol SA/mol glc and total aromatic compound yield (TACY) (including SA, DHS and QA) of 0.34 mol aromatic compounds/mol glc in 1-L fed-batch cultures using mineral broth with 15 g/L yeast extract and glucose addition to maintain a 55-170 mM concentration [4]. The effects of carbon and phosphate limitations in chemostat cultures on SA production have been studied elsewhere [15,19].

Our group has been involved in the characterization of *E. coli *strains lacking the phosphoenolpyruvate: carbohydrate phosphotransferase system (PTS^-^), such as strain PB12 (PTS^- ^glc^+^), which has been selected as an evolved strain for growth rate recovery in a chemostat with glucose fed at progressively faster rates [21,22]. This strain utilizes galactose permease (GalP) and glucokinase (Glk) to transport and phosphorylate glucose into glucose-6-P, respectively (Figure 1). In addition, most of the glycolytic and other CCM genes are upregulated in this derivative as compared to its parental strains [21-25]. Further characterization of this evolved strain has shown increased PEP availability that can be redirected into the aromatic pathway, as compared to isogenic PTS^+ ^strains. PB12 strain has been modified for the high yield production of aromatic compounds such as L-phenylalanine [26,27] and L-tyrosine [28].

In this work, we report the construction of a SA overproducing derivatives from the *E. coli *PB12 strain by inactivation of the *aroL *and *aroK *genes and expressing in plasmids different combinations of *aroG*^fbr^, *tktA*, *aroE*, and *aroB *genes. The effects of single inactivation of either pyruvate kinase (Pyk) I or II, coded respectively by *pykF *and *pykA*, were also evaluated. This strategy was used to achieve additional availability of PEP for the synthesis of aromatic compounds and SA in the *E. coli *PB12 PTS^-^glc^+^background.

## Results and discussion

### Inactivation of the genes coding for shikimate kinases I and II, and expression of the *aroG*^fbr^, *tktA*, *aroB *and *aroE *genes in plasmids in the PB12 strain background

**Figure 2:**
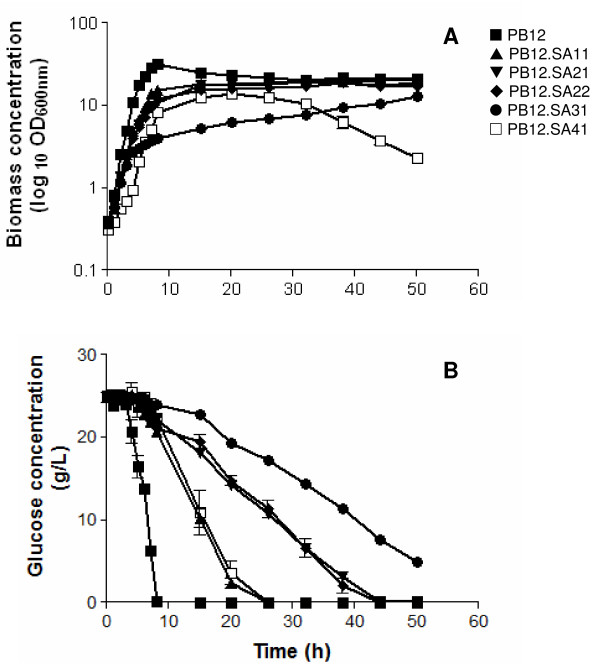
**Biomass and glucose concentrations in PB12 and SA-producing derivatives**.

**Figure 3:**
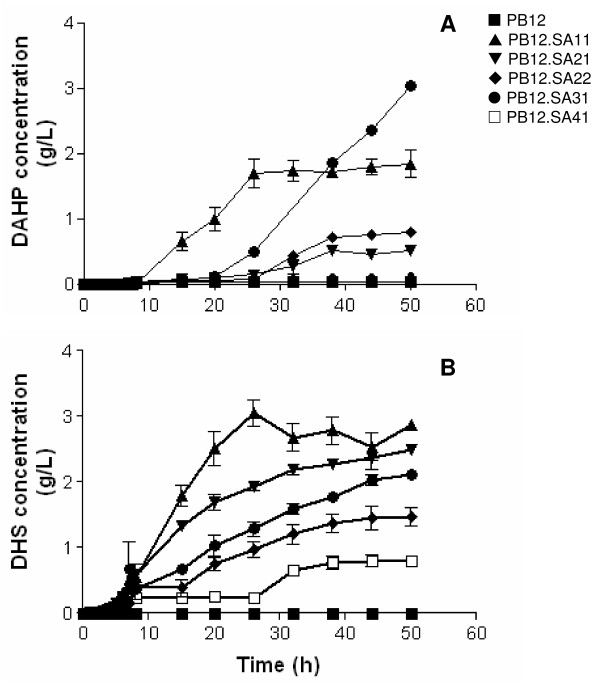
**DAHP and DHS concentrations in PB12 and SA-producing derivatives**.

The capacity of the *E. coli *PB12 (PTS^- ^glc^+^) strain to produce SA was evaluated in 500 mL batch cultures in 1 L fermentors grown in mineral broth supplemented with 25 g/L of glucose and 15 g/L of yeast extract. Specific growth rate (*μ*), glucose consumption (*qs*), SA production and yield, as well as DAHP, DHS and GA concentrations were evaluated during 50-h cultures (Table 1, Figure 2). Strain PB12 reached an OD_600 nm _of 32, after 8 h of fermentation with the consumption of 98.7% of added glucose. From this time (8 h) to the end of the fermentation (50 h), a decrease in biomass concentration was observed (Figure 2). Analysis of culture supernatants showed that as expected, strain PB12 did not accumulate DAHP (Table 2, Figure 3).

**Table 1:** Growth kinetic parameters for strain PB12 and SA-producing derivatives.

Strain/derivative	*μ*^a^ (h^-1^)	*qs*^b^ (millimol glc g DW^-1 ^h^-1^)^c^
PB12	0.48 ± 0.02	5.17 × 10^-6 ^± 4.07 × 10^-7^
PB12.SA11	0.41 ± 0.00	2.03 × 10^-6 ^± 4.79 × 10^-7^
PB12.SA21	0.42 ± 0.02*	2.5 × 10^-6 ^± 8.55 × 10 × 10^-7^*
PB12.SA22	0.42 ± 0.01*	1.93 × 10^-6 ^± 5.9 × 10 × 10^-7^*
PB12.SA31	0.32 ± 0.02	7.76 × 10^-6 ^± 9.67 × 10^-8^
PB12.SA41	0.45 ± 0.03	2.58 × 10^-6 ^± 5.39 × 10^-7^

Values are the average of two independent experiments. ^a ^*μ*, specific growth rate; ^b^*qs*, specific glucose consumption rate; ^c^DW, dry cell weight. Mean values within each column with the same superscript (*) (P < 0.05) do not differ significantly with respect to the immediate parental strain (see Methods).

**Table 2:** Aromatic metabolites production and yields determined for strain PB12 and SA-producing derivatives.

Strain	SA (g/L)	SA yield (mol SA/mol glc)	DAHP (g/L)	DHS (g/L)	GA (g/L)	TACY^1^ (mol aromatic compounds/mol glc)
PB12	ND	---	0.044 ± 0.07	ND	ND	0.00
PB12.SA11	2.82 ± 0.01	0.11 ± 0.00	1.71 ± 0.07	2.79 ± 0.21	0.21 ± 0.06	0.28
PB12.SA21	5.07 ± 0.00	0.21 ± 0.00	0.52 ± 0.00	2.49 ± 0.06*	0.14 ± 0.00	0.33*
PB12.SA22	7.05 ± 0.06	0.29 ± 0.00	0.81 ± 0.04	1.46 ± 0.14	0.08 ± 0.01	0.37*
PB12.SA31	4.35 ± 0.57	0.22 ± 0.04	3.03 ± 0.00	2.12 ± 0.02	0.23 ± 0.04	0.50
PB12.SA41	1.00 ± 0.36	0.03 ± 0.02	0.14 ± 0.00	0.79 ± 0.01	ND	0.07

Values are the average of two independent experiments. ^1^TACY, Total aromatic compound yield (combined DAHP, DHS, SA and GA molar yields); ND, Non-detected. Mean values within each column with the same superscript (*) (P < 0.05) do not differ significantly with respect to the immediate parental strain (see Methods).

**Figure 4:**
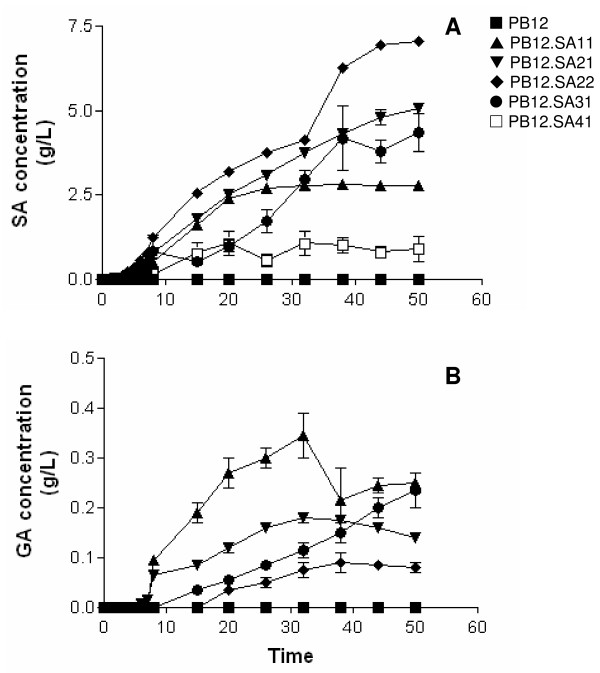
**SA and GA concentrations in PB12 and SA-producing derivatives**.

Similarly to strain PB12, cultures of strain PB12.SA11 (*aroL*^- ^strain expressing *aroG*^fbr^, *tktA *and *aroB *from two different plasmids) (see Methods, Figure 1 and Table 3) also showed an exponential growth phase during the first 8-h cultivation interval as detected for strain PB12 (Figure 2). However, a significant decrease (P < 0.05), determined by the Tukey's Honestly Significant Difference (HSD) test, was observed (see Methods) in both *μ *and *qs *values (86% and 39%, respectively), when compared to those recorded for strain PB12 (Table 1). From this moment (8 h) the strain remained stationary. DAHP, DHS, SA, and GA production was detected during the exponential growth phase (Figures 3 and 4). Interestingly, relatively constant concentration levels of all aromatic intermediates were observed after glucose was completely consumed. DAHP, DHS, and SA accumulated during the first 26 h of cultivation; thereafter their concentration remained constant at around 1.71, 2.8, and 2.8 g/L, respectively (Table 2,). SA yield on glucose was 0.11 mol SA/mol. GA concentration was lower than the other aromatic intermediates; however, as in the case of DHS, this strain produced higher GA concentrations (approximately 0.3 g/L, Table 2, Figure 4), than the other PB12 derivatives. It has been proposed that GA is formed by the oxidation of DHS into a diketo intermediate protocatehuic acid (PCA) followed by its spontaneous aromatization. Alternatively, this compound may result from the dehydration of DHS followed by hydroxylation of the intermediate PCA [29] (Figure 1). GA accumulation during SA production has not been reported in either batch or fed-batch cultures [4], but it has been detected in batch and chemostat cultures under carbon-limited conditions [15].

**Table 3:** Strains and plasmids used and developed in this work.

Strain/derivative	Relevant characteristics	Reference
*E. coli *JM101	*supE*, *thi*, Δ(*lac-proAB*), F'	[45]
*E. coli *JM101 *aroK*^-^	*E. coli *JM101 *aroK*Δ::*cm*	This work
*E. coli aroB*^-^	*E. coli *K12 strain BW25113 Δ*aroB*:*:kan *(JW3352)	[48]
*E. coli aroE*^-^	*E. coli *K12 strain BW25113 Δ*aroE*:*:kan *(JW3242)	[48]
*E. coli *PB28	PB12 Δ*pykA*::*cat *Δ*pykF*::*gen*	[31]
*E. coli *PB12	JM101 Δ(*ptsH-I-crr*)::*kan *glc^+^	[18]
PB12.SA1	PB12 Δ*aroL*	This work
PB12.SA11	PB12.SA1 pJLB*aroG*^fbr ^*tktA *pTOPO*aroB*	This work
PB12.SA2	PB12 Δ*aroL *Δ*aroK::cm*	This work
PB12.SA21	PB12.SA2 pJLB*aroG*^fbr ^*tktA *pTOPO*aroB*	This work
PB12.SA22	PB12.SA2 JLB*aroG*^fbr ^*tktA *pTOPO*aroB aroE*	This work
PB12.SA3	PB12.SA2 Δ*pykF::gen*	This work
PB12.SA31	PB12.SA3 pJLB*aroG*^fbr ^*tktA *pTOPO*aroB aroE*	This work
PB12.SA4	PB12.SA2 Δ*pykA::gen*	This work
PB12.SA41	PB12.SA4 pJLB*aroG*^fbr ^*tktA *pTOPO*aroB aroE*	This work
*E. coli *TOP10	F^- ^*mcrA *Δ(*mrr-hsd*RMS-*mcrBC*) *φ*80*lacZ*ΔM15 Δ*lacX*74 *recA*1 *ara*D139 Δ(*ara-leu*)7697 *galU galK rpsL end*A1*nupG*	Invitrogen
Plasmids		
pJLB*aroG*^fbr ^*tktA*	pJLB*aroG*^fbr ^(*aroG*^fbr ^expressed from the *lacUV5 *promoter, *lacI*q and *tet *genes (Tc^r^), pACYC184 replication origin) derivative, containing the *tktA *gene with its native promoter.	[21,47]
pCR^®^-Blunt II-TOPO^®^	P_lac _*lacZ-α *ORF T7 promoter *ccdB kan *(Km^r^) Zeocin pUC origin.	Invitrogen
pTOPO *aroB*	pCR^®^-Blunt II-TOPO^® ^containing the *aroB *gene	This work
pTOPO *aroB aroE*	pTOPO*aroB *derivative containing the *aroB *and *aroE *genes	This work

Plasmid-coded AroG^fbr ^DAHPS avoided feedback inhibition of the first reaction of the SA pathway by the phenylalanine present in the yeast extract included in the medium or produced by the cell. It has been reported that DAHPS activity *in vivo *is limited by PEP and E4P availability and that maximum specific activity of DAHPS is reached when the concentration of both intermediates is increased [13,17]. It has also been reported that the presence of a plasmid-coded copy of *tktA *(coding for transketolase I) causes an increase in E4P availability in strain PB12 [21,22,25,30-32]. In addition, it is expected that the presence of *aroB *in a multicopy plasmid (Figure 1) will reduce the possible accumulation of DAHP [3,4].

Inactivation of *aroL *gene as part of a SA production strategy has been previously described in the *E. coli *W3110 *aroL*^- ^strain (W3110 shik1) in chemostat cultures that resulted in maximum SA yields on glucose of 0.02 and 0.05 mol/mol under carbon and phosphate limited conditions that resulted in a maximum SA yield on glucose of 0.2 mol/mol and 0.05 mol/mol, respectively [15,19]. In the present study, strain PB12.SA11 yielded 0.11 mol SA/mol glc; however, maximum yields of SA on glucose of 0.27 and 0.33 mol/mol have been reported for another *E. coli *strain (PTS^- ^*glf, glk, aroF*^fbr^, *tktA, aroE, aroK*^- ^*aroL*^-^) in 1-L and 10-L fed-batch cultures, respectively [4], suggesting that the Δ*aroL *phenotype is in itself insufficient to achieve high SA yields.

Strain PB12.SA21 (*aroL*^- ^*aroK*^- ^strain expressing plasmid coded *aroG*^fbr ^*tktA *and *aroB *genes) (Figure 1 and Table 3) showed an exponential growth phase during the first 8 h fermentation interval and a stationary stage similar to what was observed for PB12 and PB12.SA11 strains (Figure 2). No significant differences (P < 0.05) were observed in *μ *and *qs *values between PB12.SA21 and PB12.SA11 strains as a consequence of the inactivation of the *aroL *and *aroK *genes (Table 1). DAHP, DHS, SA, and GA production was also detected during the exponential growth phase, but important differences were observed (Figures 3 and 4). Compared to the PB12.SA11 derivative, maximum concentrations of DAHP and GA in the PB12.SA21 strain were significantly lower (P < 0.05), whereas no significant difference (P < 0.05) was observed in the maximum concentration of DHS (Table 2, Figure 3). Furthermore, SA production was observed throughout all the process (Figure 4). After 50 h of cultivation, about 5.1 g/L of SA were detected with a yield on glucose of 0.21 mol/mol. This result represents a significant increase (P < 0.05) (80%) in both SA concentration and yield, as a consequence of the double *aroK*^- ^*aroL*^- ^mutations (Table 2, Figure 4). The concentrations of DAHP, DHS (Figure 3) and SA obtained in strain PB12.SA11, as compared to the ones recorded for the PB12.SA21 derivative, indicate an efficient flow of aromatic intermediates from DAHP to SA. However, based on the still relatively high DHS concentration observed, it appears that this strain can further convert part of the remaining DHS to improve SA concentration and yield.

Cultures of strain PB12.SA22 (*aroK*^- ^*aroL*^- ^strain expressing *aroG*^fbr^, *tktA, aroB *and *aroE *from two different plasmids) (Table 3, Figure 1) showed no significant differences (P < 0.05) in *μ *and *qs *values with respect to those for strain PB12.SA21 (Table 1). Glucose was totally consumed in both strains only after 38 h of cultivation (Figure 2). DHS was detected in a significantly (P < 0.05) lower concentration than the previous derivative and SA reached the highest concentration compared to all other derivatives (Table 2, Figure 4). At the end of the fermentation, 7.1 g/L of SA were detected with a yield on glucose of 0.29 mol SA/mol (39% increase in yield with respect to the previous derivative) and a TACY value of 0.378 mol aromatic compounds/mol glc (Table 2, Figure 4). Introduction of a copy of the *aroE *gene in the multicopy plasmid pTOPO resulted in a more efficient conversion of DHS into SA, probably as a consequence of a responsible for the synthesis of DHS from SA [3,4,15,19,20]. Accordingly, very small amounts of GA were produced during the cultivation of this strain (Table 2, Figure 4).

### Inactivation of the genes coding for pyruvate kinases I and II in the PB12SA.22 strain

Disruption of the *pykF *gene in strain PB12.SA22 generated the PB12.SA31 derivative (*aroL*^-^, *aroK*^-^, *pykF*^- ^strain expressing *aroG*^fbr^, *tktA, aroB *and *aroE *genes from two different plasmids) (Figure 1 and Table 3). Cultures of this strain showed the characteristic two-phase growth behavior observed for the previous derivatives (Figure 2), although significant (P < 0.05) differences were observed in *μ *and *qs *values as compared to the PB12.SA22 *pykF*^+ ^parental strain (Table 1). In addition, maximum biomass concentration after 8 h of fermentation was only 33% with respect to the one recorded for PB12.SA22 and, contrary to all other analyzed strains, glucose was not completely consumed after 50 h (Figure 2). DHS, GA, and specially DAHP final concentrations were higher than those obtained with the *pykF*^+ ^parental strain, whereas the final SA production and yield were lower (Table 2). Importantly, the TACY value in this strain was 0.50 mol aromatic compounds/mol glc (i.e., a 33% increment with respect to PB12.SA22), the highest yield obtained when compared to all previous PB12.SA derivatives (Table 2).

Inactivation of the *pykA *gene in strain PB12.SA22 generated the derivative PB12.SA41 (*aroL*^-^, *aroK*^-^, *pykA*^- ^strain expressing *aroG*^fbr^, *tktA, aroB *and *aroE *genes from two different plasmids) (Table 3, Figure 1). Cultures of this strain showed no-significant differences (P < 0.05) in *μ *and *qs *values with respect to those recorded for the parental strain PB12.SA22 *pykA*^+^ (Table 1). This strain reached an OD_600 nm _of 14 after 20 h of fermentation; however, an important decrease in growth was observed from this moment to the end of the fermentation (Figure 2). This strain also showed the lowest production of DAHP, DHS, and SA as compared to all other variants, and no GA was detected (Table 2, Figures 3 and 4).

Pyruvate kinase isoenzymes Pyk I and Pyk II play a key role in the glycolytic pathway, especially in overall carbon metabolism in strains lacking PTS [33,34]. Pyk activity, together with phospho-fructokinase I and glucokinase, control the carbon flux through the glycolytic pathway and catalyze the essentially irreversible trans-phosphorylation of PEP and ADP into PYR and ATP [33]. It has been previously reported that inactivation of *pykF *in strain PB12 (PTS^-^glc^+^) results in an apparently slight increase in the specific activity of Pyk A enzyme (13.5%) [34]. Likewise, carbon flux analysis in this strain has shown a flux increase through the Pyk AF enzymes in the absence of PTS as compared to the wild-type strain (JM101 PTS^+^) [23]. Furthermore, transcriptome analyses in strain PB12 and in a phenylalanine overproducing PB12 derivative have shown a slight upregulation of *pykA *with respect to the wild type strain JM101, suggesting that the overall activity of PyK isoenzymes present in the PB12 strain is sufficient to convert PEP into PYR, at least at similar rates as in JM101 [31,35]. These results suggest that single inactivation of the *pykF *or *pykA *gene could be an attractive strategy to increase the amount of PEP available for DAHP synthesis, without compromising the synthesis of PYR and its flux to acetyl-CoA.

Interruption of either the *pykF *or *pykA *gene in the *E. coli *strain PB12.SA22 demonstrated a differential role of Pyk isoenzymes in overall cellular metabolism in this strain which produces aromatic compounds. Disruption of *pykF *in strain PB12.SA31 negatively affected growth, glucose consumption, and SA accumulation with respect to the PB12.SA22 *pykF*^+ ^parental strain. Importantly, the TACY value increased to 0.50 mol aromatic compounds/mol glc in the *pykF*^- ^strain, which is 33% higher than the total yield observed for the parental strain PB12.SA22. These results suggest that *pykF *inactivation apparently increases PEP availability, which in turn is channeled into the aromatic pathway, resulting in a higher TACY value. Higher DAHP concentrations produced by the PB12.SA31 derivative also indicate that in this genetic background, DHQ synthase could be one of the limiting steps for SA production. This explanation contravenes the fact that this strain was transformed with a plasmid-carrying *aroB; *however, a previous report on the proteomic response to *pykF *inactivation in *E. coli *BW25113 strain demonstrated the upregulation of all the genes of the SA pathway, with the exception of *aroB*, during the production of aromatic amino acids [33]. Therefore, increasing the expression of the *aroB *gene, by substitution of its natural promoter for a stronger one, could be a viable strategy to improve SA concentrations in strain PB12.SA31.

Pyk activity plays a key role in cellular metabolism by connecting glycolysis with amino acid and lipid biosynthetic pathways [34,36,37]. Consequently, one remarkable characteristic of Pyk isoenzymes is their allosteric response to several effectors involved not only in central carbon metabolism but also in global cellular metabolism, among them, the glycolytic intermediate PEP [34,38,39]. It has been proposed that Pyk isoenzymes are involved in catabolite repression in *E. coli *glucose fermentations [40]; however, no information is available to correlate the specific role of individual Pyk isoenzymes in global bacterial metabolism, particularly in strains devoted to the production of aromatic compounds. Our results demonstrate that inactivation of the *pykA *gene in strain PB12.SA41 caused a negative effect on the production of aromatic compounds, probably due to an increased growth rate (Table 1). In addition, SA accumulation and TACY were substantially reduced in this strain as compared to the PB12.SA31 (*pykF*^-^) and PB12.SA22 (*pykA*^+ ^*pykF*^+^) strains. The lack of *pykF *clearly reduced *μ *and *qs *values in relation to the parental PB12.SA22 strain. In addition, glucose was not completely consumed in strain PB12.SA31 after 50 h, as compared to strain lacking *pykA*, where it was completely consumed after 25 h (Figure 2). Furthermore, the accumulation of aromatic compounds was the highest in the strain lacking *pykF*, while in the strain PB12.SA22, 37% of glucose was converted into aromatic compounds; this amount increased to 50% in strain PB12.SA31. Altogether, the result of differentially inactivating the kinases I and II suggest that the PykF isoenzyme may have a more relevant role in global cellular processes than PykA in the derivatives constructed under the growth conditions tested here, since it seems that the absence of *pykF *apparently allows higher accumulation of PEP than the absence of *pykA*. Importantly, *pykF *is apparently transcribed when growing on glucose from at least three different promoters in strains JM101 and PB12, while *pykA *is apparently only transcribed from two [41]. These results are in agreement with previous observations which suggest that PykF plays a more important role than PykA in strain JM101 (PTS^+^) and other derivative strains lacking PTS, when growing on glucose as the only carbon source [34].

## Conclusions

*E. coli *PB12 (PTS^- ^glc^+^) strain was used as the host for the synthesis of SA. The derivative PB12.SA22 was obtained by inactivation of both *aroL *and *aroK *genes, and transformed with plasmids carrying *aroG*^fbr ^*tktA, aroB*, and *aroE *genes. This strain was capable of efficiently channeling carbon from metabolites participating in the CCM into the aromatic pathway for the synthesis of SA. Fermentor cultures of PB12.SA22 strain in mineral broth complemented with 25 g/L glucose and 15 g/L yeast extract resulted in the production of 7 g/L of SA with a yield of SA on glucose of 0.29 mol/mol and a TACY of 0.38 mol aromatic compounds/mol glc. Importantly, glucose was totally consumed in strain PB12.SA22 after 48 h of fermentation. It is known that PTS^- ^strains are capable of utilizing higher concentrations of glucose (100 g/L) [42,43] and different carbon sources simultaneously with glucose [29,31]. Therefore, experiments with higher glucose concentrations, including fed-batch fermentations should be performed to increase SA concentrations. In fact, preliminary results, in which glucose concentration in the medium was increased to 100 g/L, in a 500 mL batch fermentor cultures with strain PB12.SA22, allowed the production of 14 g/L of SA (unpublished results).

Single inactivation of either the *pykF *or *pykA *gene was performed to further increase PEP availability for SA production in strain PB12.SA22. Inactivation of these genes demonstrated differential roles of Pyk isoenzymes in final growth, glucose consumption, and production of aromatic intermediates and SA. The *pykF*^- ^mutation present in strain PB12.SA31 substantially affected biomass concentration, glucose consumption, and SA production, suggesting a more important role of the PykF isoenzyme in comparison to PykA, in these growing conditions. The production of SA was reduced in this strain as compared to strain PB12.SA22; however, it is notable that TACY reached a value of 0.5 mol aromatic compounds/mol glc, which was 33% higher than the one obtained in the parental *pykF*^+ ^strain. As far as we know, there are no reports in which the utilization of a double PTS^-^, *pykF*^- ^derivative has been used to improve the production of SA and its aromatic precursors [3,4,16-18,44]. Further genetic modifications will be undertaken in this *pykF*^- ^derivative, such as the substitution of the *aroB *natural promoter for another that allows its upregulation to avoid the accumulation of the aromatic intermediate DAHP in order to increase the production of SA. In addition, carbon flux could still be further modulated by reducing the expression of *pykA *in the strain lacking *pykF*, to obtain a higher accumulation of PEP to be channeled into the SA pathway. The *pykA *gene in these *E. coli *derivatives, as mentioned, is expressed from two different promoters when glucose is utilized as the only carbon source [41]. Therefore, it could be possible to construct derivatives lacking one of these two promoters to reduce the transcription of *pykA *with the goal of increasing PEP concentration.

This study demonstrated for the first time the simultaneous inactivation of PTS and *pykF *as part of a strategy to improve SA production and its aromatic precursors in *E. coli*, with the resulting high yield of 0.5 mol aromatic compounds/mol glc.

## Methods

### Bacterial strains and plasmids

Bacterial strains and plasmids used in this work are listed in Table 3. *E. coli *PB12, a derivative of strain JM101 [45], was used as the parental strain to originate the interruptions in *aroL *and *aroK *as well as the single interruption of *pykF *or *pykA*. Amplification of target genes was performed with *Pfu *DNA polymerase (Fermentas, Glen Burnie, USA), according to recommendations by the supplier, in a GeneAmp PCR System thermocycler (Perkin Elmer Cetus, Norwalk, USA). Primer sets employed for amplification of target genes are listed in Table s1 (see Additional file 1). The size of the PCR products was determined by agarose gel electrophoresis. When required, amplicons were purified by cutting the desired band from the agarose gels and processed with a gel PCR purification kit (Marligen Biosciences, Urbana-Pike-Ijamsville, USA). The obtained derivative strains were transformed with plasmids carrying the *aroG*^fbr^, *tktA, aroB*, and *aroE *genes (see below) for the construction of SA producing strains.

#### Inactivation of the *aroL *gene

PB12.SA1 strain (*aroL*^- ^derivative) (Table 3) was obtained by the one-step inactivation procedure of chromosomal genes by PCR products [46]. Primer sets used are listed in Table s1 (see Additional file 1). The *aroL *gene was replaced by the Δ*aroL::cat *cassette. Selection was performed in chloramphenicol (Cm) containing Luria Bertani (LB) plates. Inactivation of the a*roL *gene in chloramphenicol resistant (Cm^r^) colonies was confirmed by PCR and the size of the PCR product was determined by agarose gel electrophoresis. The Cm cassette was deleted from the Δ*aroL::cat *construction, as previously described [46], to facilitate subsequent gene inactivation; the *aroL*^- ^genotype was confirmed by PCR.

#### Inactivation of the *aroK* gene

Strain PB12.SA2 (*aroL*^-^, *aroK*^- ^derivative) (Table 3) was constructed in a two-step procedure. First, the *aroK *gene of *E. coli *JM101 strain was replaced by the Δ*aroK::cat *cassette [46]. Selection was performed in Cm containing plates and the inactivation of *aroK *in Cm^r ^colonies was confirmed by PCR. Second, strain PB12.SA1 was the recipient of P1 phage lysate grown on the JM101 *aroK *strain; the *aroK*^- ^genotype was confirmed by PCR.

#### Inactivation of the *pykF* gene

PB12.SA22 strain was the recipient of P1 phage lysate of *E. coli *PB28 (Δ*pykF::gen*) strain (Table 3). Transductants were selected on gentamicin (Gm) plates and the inactivation of *pykF *in Gm^r ^colonies was confirmed by PCR; the size of the PCR product was determined by agarose gel electrophoresis. The resultant strain (*aroL*^-^, *aroK*^-^, *pykF*^- ^derivative) was named PB12.SA31.

#### Inactivation of the *pykA* gene

PB12.SA4 strain (*aroL*^-^, *aroK*^-^, *pykA*^-^derivative) (Table 3) was constructed by a modification of the one-step inactivation procedure of chromosomal genes by PCR products [46]. Briefly, template plasmids pKD3, pKD4, or pKD13, used to amplify FRT-resistance gene-FRT cassette, only allowed the use of Cm or kanamycin (Km) as selection markers [46]; however, PB12.SA2 carried both resistance genes as a consequence of previous genetic modifications [31]. For this reason, a primer set was designed (Table s1, see Additional file 1), for priming the Gm^r ^cassette flanked by the entire FRT sequence and homology regions for the *pykA *gene. The Gm^r ^cassette was amplified using chromosomal DNA from PB12.SA3 as template and the expected product was confirmed by PCR. Purified PCR products were used to inactivate *pykA *in strain JM101. Selection of the resultant Δ*pykA::gen *mutant was achieved on Gm containing plates and the inactivation of *pykA *in Gm^r ^colonies was confirmed by PCR; the size of the PCR products was determined by agarose gel electrophoresis. The Δ*pykA::gen *construction was then P1 phage transduced to PB12.SA3; Gm^r ^colonies were selected and screened.

#### Transformation of derivative strains with plasmid pJLB*aroG^fbr ^tktA*

The construction of plasmid pJLB *aroG*^*fbr *^*tktA *(Table 3) has been previously reported [21,47]; this vector was used to transform all SA producing derivative strains. Positive clones were selected by growing colonies on LB plates supplemented with tetracycline (Tet).

#### Cloning the *aroB* gene and transformation with plasmid pTOPO*aroB*

The *aroB *gene (1484 bp) was obtained by PCR using chromosomal DNA from *E. coli *JM101 strain as template and the primers Fw*aroB *and Rv*aroB *(Table s1, see Additional file 1). PCR reaction was performed with *Pfu *polymerase; the size of the PCR product was determined by agarose gel electrophoresis and cloned directly into the pCR^®^-Blunt II-TOPO^® ^vector (Invitrogen, Carlsbad, USA) leading to the construction of the pTOPO*aroB *plasmid (Table 3). This vector was used to transform competent TOP10 cells (Invitrogen) and selection was performed on 25 *μ*g/mL of zeocin-containing LB plates. Functionality of the cloned *aroB *gene was tested by restoring growth of an *aroB*^- ^*E. coli *mutant [48] in M9 minimal medium plates supplemented with zeocin, as a consequence of the complementation of the SA pathway in this mutant strain.

#### Cloning the *aroE* gene and transformation with plasmid pTOPO*aroB aroE*

The *aroE *gene (835 bp) was obtained by PCR using chromosomal DNA from *E. coli *JM101 strain as template and primers Fw*aroE *and Rv*aroE *(Table s1, see Additional file 1). PCR amplification was performed as described for the *aroB *gene; the size of the PCR product was determined by agarose gel electrophoresis. Amplified *aroE *gene and plasmid pTOPO*aroB *were both digested with *Bam *HI endonuclease. This vector was treated with calf intestine phosphatase (Fermentas) and ligated with the digested *aroB *product using T4 DNA ligase (Fermentas), transformed into TOP10 competent cells and selection was performed on zeocin-containing LB plates. Functionality of the cloned *aroE *gene was tested by restoring growth of an *aroE*^- ^*E. coli *mutant [48] in M9 minimal medium plates supplemented with zeocin.

### Cultivation media and growth conditions

Shake flask cultures inoculated with frozen stocks of each strain were performed in 125 mL baffled flasks containing 10 mL of LB supplemented with the respective antibiotics as required: 30 *μ*g/mL Km, 15 *μ*g/mL Gm, 20 *μ*g/mL Cm or 30 *μ*g/mL Tet (Table 3 shows specific antibiotic resistances). Cultures were incubated overnight in a shaker (New Brunswick Scientific, Edison, USA) at 37°C, 300 rpm. An aliquot of 150 *μ*L from each culture was used to inoculate a 250 mL baffled flask with 50 mL of fermentation medium, whose composition has been previously reported for the production of SA, and grown as described above. This medium contained 25 g/L of glucose, 15 g/L of yeast extract [4] and the required antibiotics. Biomass concentrations were determined and calculations were performed to adjust inoculum size to an OD_600 nm _of 0.35. Batch cultures were performed in duplicate in an Applikon autoclavable glass Bio Reactor (Schiedam, The Netherlands) 1 L fermentor (500 mL of working volumen of fermentation medium supplemented with the required antibiotics). This device was connected to an Applikon ADI 1010 BioController and ADI 1025 controllers to monitor temperature, pH, impeller speed and dissolved oxygen (DO). Batch fermentations were run for 50 h at 37°C, pH 7.0 (maintained by addition of 3.0% NH_4_OH). An impeller speed of no less than 500 rpm was used to maintain DO levels at 20% air saturation. Gene expression of cloned genes was induced by adding 0.1 mM IPTG at the onset of fermentation.

### Analytical procedures

Biomass concentrations were monitored every hour during the first 8 h of culture; after this point they were monitored every 6 h until the end of the fermentation. Samples (1.5 mL) were withdrawn from each reactor and cell turbidity was determined spectrophotometrically at 600 nm (Beckman DU^®^-70 Spectrophotometer, Palo Alto, USA). Samples for the determination of SA, DHS, QA, and GA were prepared by centrifuging at 12,000 rpm for 1 min (Eppendorff Centrifuge 5410, Brinkman Instruments Inc., Westubury, USA) 1 mL of fermented broth to remove cells and filtered through 0.45 *μ*M nylon membranes (Millipore, Brazil). SA, DHS, QA, and GA concentrations were determined by HPLC using a Waters system (600E quaternary pump, 717 automatic injector, 2410 refraction index, and 996 photodiode array detectors, Waters, Milford, USA), equipped with an Aminex HPX-87H column (300 × 7.8 mm; 9 *μ*m) (Bio-Rad, Hercules, USA) maintained at 50°C. The mobile phase was 5 mM H_2_SO_4_, with a flow rate of 0.5 mL/min, at 50°C. All metabolites were detected with a photodiode array detector at 210_nm_. DAHP concentrations were determined by the thiobarbituric acid assay [49]. This method does not distinguish between DAHP and DAH, so in this work, DAHP levels corresponded the sum of both compounds [26]. Glucose concentration was assessed by a biochemical analyzer (YSI 2700 Select, Yellow Springs, USA).

### Calculations

The specific glucose (S) consumption rate (*qs*) was calculated during the exponential growth phase as the differential change in S with time (t) normalized to the biomass concentration . A predetermined correlation factor (1 OD_600 _corresponded to 0.37 g/L of dry cellular weight) [50] was used to transform OD_600 _values into cell concentrations for *qs *calculation. TACY determinations were based on the combined molar yields of DAHP, DHS, SA, and GA [4].

In order to determine whether the observed differences between growth, *qs*, and aromatic intermediate production (DAHP, DHS, SA and GA) in strain PB12 and in PB12.SA derivatives were significant (P < 0.05), an analysis of variance (ANOVA) and the multiple comparison test of Tukey's Honestly Significant Difference (HSD) were performed using the XLSTAT program V2009.5.01 http://www.xlstat.com.

## Competing interests

The authors declare that they have no competing interests.

## Authors' contributions

AE and FB participated in the design of this study. AE and RC participated in the construction of Δ*aroK*, Δ*AroL*, Δ*pykF *mutants and data analysis. RC was involved in the construction of pTOPO *aroB aroE *vector and fermentations. AV participated in the construction of the Δ*pykA *mutant and fermentations. RA was responsible for the fermentations. GH performed HPLC determinations and data analysis. AE, OR, GG, and FB participated in the analysis of the results, as well as in writing and critical review of the manuscript. All authors have read and approved the manuscript.

## Supplementary Material

Additional file 1 **Table s1. Primers used in this work**. Primers used for the amplification of inactivated and cloned genes. Click here for file
