# Supplementary material for: Metabolic engineering for the production of shikimic acid in an evolved Escherichia coli strain lacking the phosphoenolpyruvate: carbohydrate phosphotransferase system
Source: Microb Cell Fact. 2010 Apr 12;9:21. doi: 10.1186/1475-2859-9-21 (PMC2873404; doi:10.1186/1475-2859-9-21)
Supplement: Additional file 1 — Table s1. Primers used in this work. Primers used for the amplification of inactivated and cloned genes. [file 1475-2859-9-21-S1.DOC]

## Table s1. Primers used in this work.

| Intended use/gene | Primer | Sequence |
| --- | --- | --- |
| Gene inactivation^a^ |  |  |
| *aroL* | *aroLcat*Fw  *aroLcat*Rv | 5´-GCG ACC TAT TGG GGA AAA CCC ACG ATG ACA CAA CCT CTT TTT CTG **TGT GTA GGC TGG AGC TGC TTC**-3´  5´-ACG TTA AGT ATA GGC GCT CGA AAA TCA ACA ATT GAT CGT CTG TGC **CAT ATG AAT ATC CTC CTT AG**-3´ |
| *aroK* | *aroKcat*Fw  *aroKcat*Rv | 5´-AAT AGT CTT AGT AGT ACC GAA AAA ATG GCA GAG AAA CGC AAT ATC **TGT GTA GGC TGG AGC TGC TTC G**-3´  5´-AGA CGA GTG TAT ATA AAG CCA GAA TTA GTT GCT TTC CAG CAT GTG **CAT ATG AAT ATC CTC CTT AG**-3´ |
| *pykA* | *pykAgen*Fw  *pykAgen*Rv | 5´-CAG TCA ACG GAG TAT TAC ATG TCC AGA AGG CTT CGC AGA **GAA GTT CCT ATA GGA TCG TGA TGT TAC GCA GCA GCA ACG AT**-3´  5´-TTT TCG CCG CAT CCG GCA ACG TAC TTA CTC TAC CGT TAA AAT **ACG GAA GTT CCT ATT TAG GTG GCG GTA CTT GGG TCG AT**-3´ |
| Cloning^b^ |  |  |
| *aroB* | Fw*aroB*  Rv*aroB* | 5’- GCTCTAGATTCAGTACGCTCAGAACGTTCA-3´^c^  5’- GGGGTACCTGAAGTTCTGGAAGCGTTGG-3’^d^ |
| *aroE* | Fw*aroE*  Rv*aroE* | 5’- CGGGATCCACAGGGGTAACATAATGGAAACC-3´^e^  5’- CGGGATCCATTCACGCGGACAATTCCTC-3’^e^ |

^a^ For gene inactivation purposes, underlined sequences correspond to homology H1 (5´- ) or H2 (3´-) priming regions for target genes, bold sequences correspond to FRT and antibiotic resistance cassette priming site [46]. ^b^For cloning purposes, underlined sequences correspond to restriction sites: ^c^ *Xba*I; ^d^ *Kpn*I; ^e^ *Bam*HI
